# Supplementary material for: Automated analysis of bacterial flow cytometry data with FlowGateNIST
Source: PLoS One. 2021 Aug 18;16(8):e0250753. doi: 10.1371/journal.pone.0250753 (PMC8372958; doi:10.1371/journal.pone.0250753)
Supplement: Attachment — Submitted filename: Response to Reviewers.docx [file pone.0250753.s002.docx]

Point-by-Point Response:

Reviewer #2:

1. Please clarify if the analysis memory is limited.

This clarification was added (lines 177-179 in the revised manuscript):

“The number of analysis memory results that can be saved is limited only by the capacity of the system where the flow cytometry data is stored.”

2. Please clarify if FlowGatesNIST can be open in any operative system.

This clarification was added (lines 71-72 in the revised manuscript):

“FlowGateNIST can be installed and used on any computer with an operating system capable of running Python 3.”

3. Please clarify if FlowGatesNIST can be open in any internet browser.

Python packages are not normally run directly in an internet browser. But Jupyter Notebooks can be used to run FlowGateNIST (and other Python packages) within a web browser. An appropriate clarification was added (lines 72-74 in the revised manuscript):

“In addition, FlowGateNIST can be run with Jupyter Notebooks [13] within most web browsers to provide a record of the analysis for each dataset.”

4. The FlowCal package inspires FlowGatesNIST, and you provided essential technical information in the result section. However, please include a paragraph to be more explicit (and easier) about the differences between this new package and FlowCal. This new information should be writing thinking in the final user, who usually is biomedical researchers or medical technicians.

That additional information on the difference between FlowGateNIST and FlowCal (lines 101-103 in the revised manuscript):

“Apart from the use of Pandas DataFrames, the primary difference between FlowGateNIST and FlowCal is that FlowGateNIST requires the measurement of blank samples, which it uses for automated cell gating (see below).”
